# Safety and efficacy of intensive vs. guideline antiplatelet therapy in high‐risk patients with recent ischemic stroke or transient ischemic attack: rationale and design of the Triple Antiplatelets for Reducing Dependency after Ischaemic Stroke (TARDIS) trial (ISRCTN47823388)

**DOI:** 10.1111/ijs.12538

**Published:** 2015-06-16

**Authors:** The TARDIS Trial Investigators

**Keywords:** acute stroke, aspirin, clopidogrel, dipyridamole, randomized controlled trial, transient ischemic attack

## Abstract

**Rationale:**

The risk of recurrence following a stroke or transient ischemic attack is high, especially immediately after the event.

**Hypothesis:**

Because two antiplatelet agents are superior to one in patients with non‐cardioembolic events, more intensive treatment might be even more effective.

**Sample size estimates:**

The sample size of 4100 patients will allow a shift to less recurrence, and less severe recurrence, to be detected (odds ratio 0·68) with 90% power at 5% significance.

**Methods and design:**

Triple Antiplatelets for Reducing Dependency after Ischaemic Stroke (ISRCTN47823388) is comparing the safety and efficacy of intensive (combined aspirin, clopidogrel, and dipyridamole) vs. guideline antiplatelet therapy, both given for one‐month. This international collaborative parallel‐group prospective randomized open‐label blinded‐end‐point phase III trial plans to recruit 4100 patients with acute ischemic stroke or transient ischemic attack. Randomization and data collection are performed over a secure Internet site with real‐time data validation and concealment of allocation. Outcomes, serious adverse events, and neuroimaging are adjudicated centrally with blinding to treatment allocation.

**Study outcome:**

The primary outcome is stroke recurrence and its severity (‘ordinal recurrence’ based on modified Rankin Scale) at 90 days, with masked assessment centrally by telephone. Secondary outcomes include vascular events, functional measures (disability, mood, cognition, quality of life), and safety (bleeding, death, serious adverse events).

**Discussion:**

The trial has recruited more than 50% of its target sample size (latest number: 2399) and is running in 104 sites in 4 countries. One‐third of patients presented with a transient ischemic attack.

## Introduction and rationale

Following stroke or transient ischemic attack (TIA), the risk of recurrence is very high over the first few hours and days, reaching 10·3% by three‐months 1, 2. Risk then declines and totals about 40% by five‐years. Importantly, recurrent strokes are usually more severe than first events and so are more likely to lead to dependency, cognitive impairment and dementia, depression, poor quality of life, and need for long‐term institutional care 3, 4.

The long‐term risk of recurrence can be reduced, but not abolished, with lifestyle changes (reducing weight, saturated fat, salt and high alcohol intake, and stopping smoking) and evidence‐based and cost‐effective clinical interventions including lowering blood pressure (BP) (all stroke and TIA) and lipids (ischemic stroke and TIA), and carotid endarterectomy (large artery ischemic stroke and TIA) 1, 2, 5, 6, 7, 8, 9, 10. While oral anticoagulants are established therapy after cardioembolic stroke and TIA 11, 12, 13, 14, the majority of patients with acute and chronic ischemic stroke or TIA need antiplatelets 15, 16, 17, 18, 19, 20, 21, 22, 23.

Antiplatelet therapy for acute ischemic stroke is based on aspirin alone as a result of the IST‐1 and CAST mega‐trials 17, 18, although the effect size for improving functional outcome was small (absolute risk reduction ∼1·1%), mostly explained by aspirin reducing early recurrence. Until recently, the acute treatment of TIA had not been investigated. Early and short‐term use of two agents appears to be superior to monotherapy, as suggested by observational studies (EXPRESS, SOS 1, 2), small trials (FASTER, EARLY) 24, 25, and a post hoc subgroup analysis of the PRoFESS mega‐trial 26. These findings were strengthened by the large CHANCE trial that showed that the combination of aspirin + clopidogrel was superior to aspirin alone in reducing stroke recurrence 23. Indeed, it appears in meta‐analyses that any pair of antiplatelets is superior to any single agent 27, 28. A potential advantage of multi‐antiplatelet therapy is that it will help cover treatment resistance seen with monotherapy for either aspirin or clopidogrel 29, 30, 31.

The situation in acute stroke and TIA differs from chronic stroke (long‐term secondary prophylaxis) where dual therapy with aspirin + dipyridamole reduced events by 23% in comparison with aspirin or dipyridamole alone without increasing the risk of bleeding (ESPS‐2, ESPRIT) 19, 21. However, dual therapy based on aspirin + clopidogrel was not superior to monotherapy with either agent alone (CHARISMA, MATCH) 32, 33. In MATCH, dual therapy caused more bleeding than clopidogrel alone but without a significant reduction in recurrence 33, 34.

If dual therapy is more effective at preventing recurrence than monotherapy for acute prophylaxis, then intensive therapy with triple antiplatelets (combined aspirin + clopidogrel + dipyridamole) may be better still, provided the risk of recurrence is high and bleeding does not become excessive. We have performed a series of ‘proof‐of‐mechanism’ and ‘proof‐of‐concept’ laboratory studies and clinical trials investigating this approach 35, 36, 37, 38, 39. *In vitro* studies starting in 2000 found that triple therapy was most effective in inhibiting platelet aggregation, platelet–leucocyte conjugation, and leucocyte activation 35, 36, 37. In multiway crossover phase I and II trials, short‐term administrations of mono (aspirin, clopidogrel, or dipyridamole), dual (combinations of aspirin and clopidogrel, aspirin and dipyridamole, or clopidogrel and dipyridamole), and triple (combined aspirin, clopidogrel, and dipyridamole) antiplatelet therapy were compared; the combination of aspirin and clopidogrel, with or without dipyridamole, was most potent in inhibiting platelet function *ex vivo* in both normal volunteers and participants with previous stroke/TIA 38, 39. (Of note, the platelet function tests used in these studies are relatively insensitive to the intracellular effects of dipyridamole.) In the only parallel group trial of intensive/triple therapy in participants with stroke, we found that combined aspirin, clopidogrel, and dipyridamole (vs. aspirin alone, chosen because it was the UK standard of care at the time) was feasible to administer in a pilot trial for up to 24 months 40. However, the trial was stopped early on publication of ESPRIT 21 confirming the superiority of combined aspirin and dipyridamole over aspirin alone. There was a non‐significant trend to increased bleeding with triple antiplatelet therapy vs. aspirin alone. Although unintended, the participants were at low risk of recurrence (young/recruited months after the event/many lacunar strokes) 40, a problem also seen in MATCH and CHARISMA 32, 33. The study concluded that future trials of combined aspirin, clopidogrel, and dipyridamole needed to target participants at high risk of recurrence and for a short treatment duration to minimize bleeding, so that benefit is likely to outweigh hazard. Clinical use of triple antiplatelet therapy has also been reported in a case series 41.

The TARDIS trial was designed to build on these laboratory and clinical studies and aims to test the overall safety and efficacy of intensive antiplatelet therapy with three agents in comparison with guideline treatment.

## Primary research question

Is intensive antiplatelet therapy (combined aspirin, clopidogrel, and dipyridamole) safe and effective in reducing recurrence and its severity at three‐months, as compared with guideline antiplatelet therapy (clopidogrel, or combined aspirin and dipyridamole), when given acutely after stroke or TIA for one‐month?

## Methods

¶ refers to a change from the current Protocol version 1·5 (downloadable from http://www.nets.nihr.ac.uk/projects/hta/1010424).

### Design

TARDIS is an international collaborative multicenter parallel‐group prospective randomized open‐label blinded‐end‐point phase III controlled trial.

### Patient population

Inclusion criteria:

- Age ≥50 years
- Event to randomization ≤48 h (24–48 h if thrombolysed)
- Index event is a TIA (defined in supplement of Statistical Analysis Plan 42) with:
  ○  Resolved limb weakness and/or dysphasia
  ○  Duration 10 min to <24 h
  ○  ABCD2 score ≥4; AND/OR crescendo TIA; AND/OR already on dual antiplatelet therapy
- Index event is a non‐cardioembolic ischemic stroke with:
  ○  Ongoing limb weakness OR ongoing facial weakness with resolved limb weakness; AND/OR dysphasia; AND/OR ongoing isolated hemianopia (with positive neuroimaging evidence showing ischemic stroke in occipital lobe); AND duration ≥one‐hour
  ○  Resolved limb weakness; AND/OR dysphasia; AND duration >24 h after onset (i.e. resolution between 24 h and randomization)
- Willing and able to provide written informed consent; proxy consent is acceptable if patients are dysphasic or confused, in accordance with the practice of the local site

Exclusion criteria:

- Isolated sensory symptoms, facial weakness, or vertigo/dizziness
- Isolated hemianopia without positive neuroimaging evidence
- Intracranial haemorrhage
- Baseline neuroimaging shows intracranial haemorrhage or parenchymal haemorrhagic transformation (PH 1 or 2) of infarct, subarachnoid haemorrhage, or other non‐ischemic cause for symptoms
- Presumed cardioembolic stroke (e.g. history of current atrial fibrillation (AF), myocardial infarction <three‐months)
- Contraindications to, or intolerance of, aspirin, clopidogrel, or dipyridamole
- Definite need for aspirin, clopidogrel, or dipyridamole individually or in combination (e.g. aspirin and clopidogrel for recent myocardial infarction (MI)/acute coronary syndrome)
- Definite need for full dose oral (e.g. apixaban, dabigatran, rivaroxaban, warfarin) or medium to high dose parenteral (e.g. heparin) anticoagulation
- Definite need for glycoprotein IIb/IIIa inhibitor
- No enteral access
- Pre‐morbid dependency [modified Rankin Scale (mRS) > 2]
- Severe high BP (BP > 185/110 mmHg)
- Haemoglobin <100 g/l
- Platelet count <100 × 10^9^/l or >600 × 10^9^/l
- White cell count <3·5 × 10^9^/l or >30 × 10^9^/l
- Major bleeding within one‐year (e.g. peptic ulcer, intracerebral haemorrhage)
- Planned surgery in next three‐months (e.g. known need for carotid endarterectomy)
- Concomitant acute coronary syndrome [e.g. ST segment elevation myocardial infarction (STEMI) or non‐STEMI (NSTEMI)]
- Stroke secondary to a procedure (e.g. carotid or coronary intervention)
- Coma [Glasgow Coma Scale (GCS) < 8]
- Non‐stroke life expectancy <six‐months
- Known dementia
- Women of childbearing potential, pregnant, or breastfeeding
- Geographical or other factors that may interfere with follow‐up
- Patients who have not had post‐thrombolysis neuroimaging
- Patients may be enrolled concurrently into observational studies or non‐drug/device trials.

### Baseline measures

Baseline demographic details (age, gender, race‐ethnicity), pre‐morbid mRS, clinical details (syndrome 43), stroke severity [National Institutes of Health Stroke Scale, (NIHSS) 44 ], BP, full blood count, and electrocardiogram (ECG) are determined after consent/assent and before randomization.

Neuroimaging – computerized tomography (CT) or magnetic resonance (MR) scanning – is performed for patients with ischemic stroke to exclude intracranial haemorrhage and non‐stroke diagnoses. If thrombolysis is performed, CT/MR must be undertaken afterward and prior to randomization to exclude haemorrhagic transformation. Patients presenting with a TIA do not have to have a CT/MR as this reflects routine practice at many stroke centers. Patients with cerebral events that occur during treatment must also be re‐scanned to identify potential secondary bleeding. Local site reporting of scans is recorded; all scans are also uploaded over the Internet for independent adjudication using a validated structured classification system [as used in IST‐3 and Efficacy of Nitric Oxide in Stroke trial (ENOS)] 45, 46, 47, 48 and masked to treatment.

At baseline and day 7 ± 1, optional research blood samples may be taken for substudies involving biomarkers and genetics; some samples are centrifuged to collect plasma and serum, and then frozen.

### Randomization

All randomization, data collection, and serious adverse event (SAE) and CT adjudication are performed over a secure password‐protected and data‐encrypted Internet website: www.tardistrial.org. Patients are randomized in real time with:

Stratification on:

- Index event: stroke/TIA
- Country

Minimization on key prognostic baseline factors:

- Age: ≤70 vs. >70 years
- Gender: female, male
- Pre‐morbid mRS: 0, >0
- Time, stroke/TIA to randomization: 24 vs. <24 h
- Number of antiplatelets before index event: 0/1, 2
- Clinical syndrome: lacunar (LACS/POCS), cortical (PACS/TACS) 49
- Systolic BP: ≤160, >160 mmHg
- Gastro‐protection: yes, no
- Use of low dose heparin: no, yes

Additional minimization is performed if the index event is an ischemic stroke:

- NIHSS: 0–3, >3
- Treatment with alteplase: yes, no

Additional minimization is performed if the index event is a TIA:

- ABCD2 score: 0–5, >5
- Number of TIAs in last week: 0/1, >1

Simple randomization:

- In 5% of patients

Stratification and minimization allow for improved matching at baseline, stratification allows variable categories to be treated as trials in their own right, minimization increases statistical power 50, and simple randomization reduces predictability.

### Investigational medicinal products

**Figure 1:**
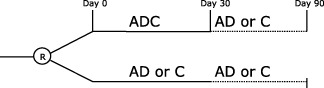
Treatment (day 0–30) and follow‐up (to day 90). A, aspirin; C, clopidogrel; D, dipyridamole.

Trial interventions are given open label for one‐month (28 or 30 days depending on treatment pack size, to cover the period of maximum risk of recurrence but minimize bleeding) and comprise (Fig. 1):

- Aspirin: loading dose 300 mg 51 then 50–150 mg daily; by oral, nasogastric tube (NGT), or rectal route
- Clopidogrel: loading dose 300 mg 52 then 75 mg daily; by oral or NGT route
- Dipyridamole: 225–450 mg in divided doses; by oral (as 200 mg extended release capsules twice daily 53, or as tablets three to four times daily) or NGT (as suspension or crushed tablets three to four times daily) route

Treatment groups comprise:

- Intensive/triple antiplatelets (active): Combined aspirin, clopidogrel, and dipyridamole
- Guideline/dual or mono antiplatelet(s): Combined aspirin and dipyridamole, or clopidogrel alone [monotherapy was added following a change in UK National Institute for Health and Care Excellence (NICE) guidance 54]

Sites choose in advance which guideline treatment regimen they wish to use, a choice that is made separately for ischemic stroke (IS) and TIA:

- Aspirin + dipyridamole, or clopidogrel (1:1)
- Aspirin + dipyridamole
- Clopidogrel

The principal investigator (PI) may change the choice of comparator at any stage during the trial with 48‐h notice, that is, a change cannot influence treatment for a patient who is in the process of being enrolled.

Antiplatelet agents may be administered after the index stroke/TIA event and before randomization as follows:

- Aspirin may be given after the index event and prior to randomization in any potential trial participant.
- Clopidogrel is only allowed if the patient may receive it as part of the trial according to the PI's choice of comparator, that is, the patient can only be randomized to intensive antiplatelets vs. clopidogrel alone.
- Dipyridamole (in combination with aspirin) is only allowed if the patient may receive it as part of the trial according to the PI's choice of comparator, that is, the patient can only be randomized to intensive antiplatelets vs. combined aspirin and dipyridamole.
- If the patient is given a ‘confounding’ antiplatelet after their event and before randomization, the patient may still be included but randomization will then only involve the appropriate comparison to prevent confounding of treatment.

Patients who have received combined clopidogrel and aspirin, clopidogrel and dipyridamole, cilostazol (whether singly or in combination), or triflusal (whether singly or in combination) are excluded from the trial.

Study drugs may be stopped around procedures that become necessary after enrollment; trial drugs should be re‐started as soon as possible after the procedure once clinically appropriate. Participants can be withdrawn from therapy either at their own request, for safety reasons, or if unacceptable adverse events develop. After the 28–30‐day treatment period, participants are expected to return to guideline antiplatelet therapy as recommended by local, national, and international guidelines. Patients are also offered standard ‘best care’ prophylaxis, including lifestyle advice, BP and lipid‐lowering drugs, and carotid endarterectomy (as necessary).

### Primary outcome

The primary outcome is the frequency and severity of recurrent strokes and TIA in participants who have a recurrent event, with assessment at day 90. Severity is measured using a six‐level ordered categorical scale that incorporates the mRS:

- Fatal stroke/severe non‐fatal stroke (mRS 4 or 5)/moderate stroke (mRS 2 or 3)/mild stroke (mRS 0 or 1)/TIA/no stroke‐TIA¶

Ascertainment of recurrent events, and mRS, is determined centrally by telephone by a trained assessor who is masked to outcome at day 90 ± 7. To ensure recurrent events are identified, corroborating information is sought from the general practitioner and recruiting hospital site.

The effect of the intervention on the primary outcome will be performed within the following subgroups:

(**a**)  Geographical region: UK, other ¶
(**b**)  Age: ≤70 years, >70 years ¶
(**c**)  Gender: female, male
(**d**)  mRS: 0, >0 ¶
(**e**)  Index event: ischemic stroke, TIA
(**f**)  Stroke/TIA syndrome: LACS, POCS, PACS, TACS 49 ¶
(**g**)  Stroke/TIA etiology: small vessel disease (SVD), large artery disease (LAD), other ¶
(**h**)  NIHSS (stroke only): 0–3, >3 ¶
(**i**)  ABCD2 score (TIA only): 0–5, >5 ¶
(**j**)  Crescendo TIA (TIA only): no, yes ¶
(**k**)  Number of antiplatelet agents at baseline: 0, 1, 2 ¶
(**l**)  Type of comparator: AD, C, either ¶
(**m**)  Systolic BP: ≤140, 141–160, >160 mmHg ¶
(**n**)  Time, event to randomization >24, 12·1–24, ≤12 h ¶
(**o**)  Use of low dose heparin: no, yes ¶
(**p**)  Treated with alteplase prior to randomization (stroke only): yes, no
(**q**)  Gastroprotection: yes, no ¶
(**r**)  Carotid stenosis (ipsilateral ≥50%): no, yes ¶
(**s**)  Old lesion on baseline neuroimaging: no, yes ¶

### Secondary, bleeding, and safety outcomes

Investigators assess secondary outcomes at days 7 ± 1 and 35 ± 3, and on discharge from hospital (if admitted). The National Coordinating Centre assesses secondary outcomes at day 90 ± 7 by a telephone call between the patient (or carer) and an assessor blinded to treatment. Reported outcomes (stroke, MI) and SAEs are adjudicated by a member of the independent adjudicator panel who is blinded to treatment.

Day 7 ¶

(**a**)  Headache that required treatment or led to discontinuation
(**b**)  Recurrent stroke or TIA
(**c**)  Impairment (NIHSS, including death)
(**d**)  Neurological deterioration (increase in NIHSS by four points or more)
(**e**)  Composite vascular event
(**f**)  Venous thromboembolism
(**g**)  Haemoglobin
(**h**)  Bleeding
(**i**)  SAEs

Day 35 (end of treatment)

(**a**)  Headache that required treatment or led to discontinuation
(**b**)  Recurrent stroke or TIA
(**c**)  Impairment (NIHSS, including death)
(**d**)  Neurological deterioration (increase in NIHSS by four points or more)
(**e**)  Composite vascular
(**f**)  Myocardial infarction
(**g**)  Venous thromboembolism
(**h**)  Haemoglobin
(**i**)  Bleeding
(**j**)  SAEs

Hospital discharge (collected at discharge or if death in hospital)

(**a**)  Length of stay in hospital
(**b**)  Discharge disposition (death/institution/home)

Day 90 (end of follow‐up)

(**a**)  Death: time to death (censored at 110 days) and by what cause
(**b**)  Composite vascular
(**c**)  Myocardial infarction 55
(d)  Venous thromboembolism
(**e**)  Barthel Index (BI)
(**f**)  Dead or disabled (BI < 60)
(**g**)  Quality of life/Health Utility Score [derived from European Quality of Life‐5 Dimensions (EQ‐5D)] 56 Δ
(**h**)  Quality of life [European Quality of Life Visual Analog Scale (EQ‐VAS)] Δ
(**i**)  Telephone‐Mini‐Mental State Examination Δ
(**j**)  Telephone Interview Cognition Scale‐Modified Δ
(**k**)  Verbal fluency (animal naming over one‐minute) Δ
(**l**)  Zung Depression Scale (mood) 57 Δ
(**m**)  Disposition (death/institution/home)
(**n**)  Bleeding – by site and severity 58
(**o**)  SAEs – by time, type, site, and severity 21

Δ will not be collected if carer answers questions without recourse to participant.

### Sample size

TARDIS was designed with a start‐up phase (funded by British Heart Foundation, and assessing safety, feasibility, and tolerability) and a main phase (funded by Health Technology Assessment, and assessing safety and efficacy).

The null hypothesis (H_0_) is that intensive antiplatelets will not alter the frequency and severity of stroke/TIA in participants with previous ischemic stroke or TIA. The alternative hypothesis is that the frequency and severity of stroke/TIA differ between those participants randomized to intensive vs. guideline antiplatelets. A total sample size 59, 60 of 4100 (2050 per group) participants with ischemic stroke or TIA is required, assuming:

- Overall significance (alpha) = 0·05
- Power (1‐beta) = 0·90
- Odds ratio of 0·68 (equivalent to an odds ratio of 0·57 and relative risk reduction = 0·31 for binary stroke)
- Distribution in outcome based on recurrent stroke and its severity using mRS (based on data from *n* = 1460 participants with a final outcome):
  ○  Fatal stroke, 0·55%/mRS 4 or 5, 0·55%/mRS 2 or 3, 1·30%/mRS 0 or 1, 1·23%/TIA, 3·22%/no event, 93·15%
- Treatment crossovers = 5·0%
- Losses to follow‐up = 2%
- Reduction of 20% for baseline covariate adjustment 61

### Statistical analyses

Analyses will be performed by intention‐to‐treat using binary logistic regression for binary outcomes, ordinal logistic regression for ordered categorical variables (including the primary outcome), multiple regression for continuous variables, and Cox proportional hazards regression for time‐to‐event data. Analyses will be adjusted for stratification and minimization factors. Detailed analysis plans are given in the Statistical Analysis Plan.

### Study organization and oversight

TARDIS is an independent academic trial performed by an international collaborative group. The Trial Steering Committee provides oversight and strategic input, and comprises independent members, grant applicants, and patient, sponsor, and funder representatives; it meets twice yearly. An International Advisory Committee meets annually and provides advice on national issues including recruitment and follow‐up. The Trial Management Committee runs the trial on a day‐to‐day basis and is based at the TARDIS Trial Coordinating Centre in Nottingham. A National Coordinating Centre and national coordinator are based in each participating country. Outcomes, SAEs, and brain imaging are adjudicated by trained assessors masked to treatment assignment.

The independent Data Monitoring Committee reviews unblinded data twice yearly in respect of safety and efficacy, and review recruitment, baseline data, balance in baseline factors between the treatment groups, completeness of data, compliance to treatment, co‐administered treatments, outcome by subgroups, SAEs (both adjudicated and unadjudicated), and protocol violations. They also take findings in the context of other published evidence.

### Research governance

TARDIS is conducted in accordance with the ethics and principles enshrined in the Declaration of Helsinki and good clinical practice, and is run in accordance with the UK Medicines for Human Use Regulations and Health Research Governance Framework. The management of personal data adheres to the UK Data Protection Act (1998). The trial has approval from the Medicines and Healthcare Products Regulatory Agency (reference 03057/0027/001‐0001, date 17/10/2008), Eudract number 2007‐006749‐21, and National Research Ethics Committee (Reference 08/H1102/112, date 9/1/2009). All sites have local Research Ethics Committee (REC) and NHS Research and Development (R&D) approvals. The trial is registered with Current Controlled Trials (ISRCTN47823388) and has been adopted by the UK NIHR Stroke Research Network, and endorsed by the Australasian Stroke Trials Network.

## Summary and conclusions

TARDIS is addressing a key issue in the management of patients with acute stroke and TIA, namely the safety and efficacy of short‐term intensive (combined aspirin, clopidogrel, and dipyridamole) vs. guideline antiplatelet therapy. The primary outcome is the frequency and severity of stroke recurrence; TARDIS is the first trial to use this novel end‐point. The sample size of 4100 patients means that a modest but worthwhile clinical effect can be detected with high statistical power (90%); to date, 2399 patients have been recruited from 104 sites in 4 countries, with one‐third presenting with a TIA. A positive trial would mean that triple antiplatelet therapy could be introduced rapidly into clinical practice as the drugs are already licensed, readily available, and inexpensive. We invite centers from around the world to join this important collaborative international venture.

## Supporting information


**Appendix S1.** Full list of acknowledgements.
