# Supplementary material for: Safety and efficacy of intensive vs. guideline antiplatelet therapy in high‐risk patients with recent ischemic stroke or transient ischemic attack: rationale and design of the Triple Antiplatelets for Reducing Dependency after Ischaemic Stroke (TARDIS) trial (ISRCTN47823388)
Source: Int J Stroke. 2015 Jun 16;10(7):1159–65. doi: 10.1111/ijs.12538 (PMC4855643; doi:10.1111/ijs.12538)
Supplement: Supplementary file 1 — Appendix S1. Full list of acknowledgements. [file IJS-10-1159-s001.pdf]

Acknowledgements

Writing Committee*

Kailash Krishnan, Maia Beridze, Hanne Christensen, Rob Dineen, Lelia Duley, Stan Heptinstall, Marilyn James, Hugh S. Markus, Stuart Pocock, Annemarei Ranta, Thompson Robinson, Nikola Sprigg, Graham Venables, Philip Bath

Trial Steering Committee (TSC)

Independent members: Helen Rodgers (Newcastle, UK; independent chair/stroke physician), Ahamad Hassan (Leeds, UK; independent stroke neurologist), Christine Roffe (Stoke-on-Trent, UK; independent expert/stroke physician), Craig Smith (Salford, UK; independent stroke physician), William Toff (Leicester, UK; independent trialist)

Grant applicants: Philip Bath (Nottingham, UK; chief investigator/stroke physician), Rob Dineen (Nottingham, UK; neuroradiologist), Lelia Duley (Nottingham, UK; trialist), Stan Heptinstall (Nottingham, UK; platelets), Marilyn James (Nottingham, UK; health economics), Hugh S. Markus (Cambridge, UK; neurologist), Stuart Pocock (London, UK; biostatistician), Thompson Robinson (Leicester, UK; stroke physician), Nikola Sprigg (Nottingham, UK; co-chief investigator), Graham Venables (Sheffield, UK; neurologist)

Participant representatives: Chibeka Kasonde (Nottingham, UK; from 2014), Ossie Newell (Nottingham, UK; to 2014)

Sponsor representative: Angela Shone (University of Nottingham)

International Advisory Committee (IAC)

Philip Bath (UK, chair), Maia Beridze (Georgia), Hanne Christensen (Denmark), Annemarei Ranta (New Zealand)

Trial Management Committee (TMC, Nottingham)

Sally Utton (senior trials manager, chair –2014), Hayley Foster (trial manager, chair 2014–), Philip Bath (chief investigator), Nikola Sprigg (co-chief investigator), Margaret Adrian (UK coordinator), Harriet Howard (UK coordinator, 2014–), Tanya Payne (UK coordinator), Michael Stringer (UK coordinator, 2014–), Alice Durham (international coordinator, 2013–14), Sarah Grant (international coordinator, 2013–14), Joanne Keeling (outcome coordinator, 2013–), Kailash Krishnan (medic, 2012–), Katie Robson (statistician, 2014), Lisa Woodhouse (statistician, 2014–), Dawn Hazle (data and imaging, 2012–), Wim Clarke (finance), Lauren Dunn (secretary, 2013–14), Yvonne Smallwood (secretary)

Independent Data Monitoring Committee

Ian Ford (Glasgow, UK; chair), Cathie Sudlow (Edinburgh, UK), Matthew Walters (Glasgow, UK), Didier Leys (Lille, France)

Statistical support (Nottingham, UK; non-voting): Samir Mehta (to 2013), Wei Tan (from 2013)

Outcome/event adjudicators (outcome, SAEs)

Nikola Sprigg (Nottingham, UK; chair), Marc Randall (Leeds, UK, 2014–)

Neuroimaging adjudicators

Rob Dineen (Nottingham, UK; chair), Alessandro Adami (Verona, Italy), Jennifer Becker (Arizona, USA), Lesley Cala (Perth, Australia), Ana Casado (Edinburgh, UK), Panos Koumellis (Nottingham, UK)

Platelet substudy

Stan Heptinstall, Sue Fox, Jane May (Nottingham, UK)

Programming/database management

Richard Dooley, Lee Haywood, Liz Walker (Nottingham, UK)
